# From Itch to Access: Psychodermatological Care Challenges and the Promise of Telehealth

**DOI:** 10.3390/jcm14061993

**Published:** 2025-03-15

**Authors:** Julia Rümmelein, Christiane Brockes, Christian Greis

**Affiliations:** 1 Clienia Schlössli AG Private Hospital for Psychiatry and Psychotherapy, 8618 Oetwil am See, Switzerland; 2 Clinical Telemedicine, University Hospital Zurich, 8091 Zurich, Switzerland; christiane.brockes@alcare.ch; 3 Department of Dermatology, University Hospital Zurich, 8091 Zurich, Switzerland; christian.greis@usz.ch

**Keywords:** pruritus, psychodermatology, telemedicine, digitalization, ehealth, skin diseases

## Abstract

**Background/Objectives:** Pruritus is a prominent symptom of chronic inflammatory skin diseases and significantly affects quality of life. Psychological stress can exacerbate pruritus and worsen skin conditions, yet psychological aspects are often insufficiently addressed in clinical routine. While psychodermatological treatments are becoming more available in German-speaking countries, they are mostly confined to specialized clinics, limiting access for many patients. This study aims to explore the unmet needs of patients with chronic inflammatory skin diseases in German-speaking regions and assess the potential role of telemedicine in bridging existing psychodermatological care gaps. **Methods:** Patients with chronic inflammatory skin diseases were invited via the Network of People with Autoimmune Diseases to participate in free video consultations with a licensed psychotherapist. Quantitative data on disease, pruritus, and distress were analyzed alongside qualitative feedback from consultations and surveys. **Results:** Of 174 individuals who received the newsletter, 124 opened it, and 16 engaged with the scheduling link. Over one month, five patients (mean age 40.4 years, all female) participated in psychodermatological video consultations. All had chronic inflammatory skin diseases and were under dermatological care but felt insufficiently treated by dermatological approaches alone. Barriers to multimodal care included lack of awareness, distance to specialized clinics, and long waiting times. Three participants reported pruritus with an average intensity of 75/100 on a Visual Analog Scale (VAS). Psychological factors were identified as significant contributors to pruritus by all participants. Post-consultation, 4/5 of participants completed a survey, reporting high levels of distress (average 74/100 VAS) and favoring online or hybrid treatment options. **Conclusions:** Dermatological treatment alone often fails to address psychological aspects in patients with chronic inflammatory skin diseases. These findings emphasize the need for integrated dermatological and psychological treatment, with telemedicine offering a valuable avenue to improve access and foster interdisciplinary collaboration.

## 1. Introduction

Pruritus, or itch, is a highly prevalent and multifaceted condition affecting approximately 40% of the general population, with individuals suffering from inflammatory skin diseases being particularly vulnerable to its debilitating effects [1,2]. The phenomenon of itch extends beyond mere sensory perception, encompassing complex dimensions of cognition, motivation, and emotion, further underlining its intricate nature [3]. Psychological factors, such as stress, are frequently identified as major triggers and amplifiers for itch, with their impact particularly pronounced in various pruritic conditions. For instance, psychological stress has been reported to aggravate itch in 71% of patients with atopic dermatitis [4] and in 55–71% of patients with psoriasis [5,6].

Stress can not only trigger the onset of chronic itch in individuals who are predisposed, but also exacerbate pruritus. In separate studies, a significant proportion of patients with atopic dermatitis (64%) and psoriasis (72.5%) reported experiencing at least one stressful life event in the month preceding an exacerbation of their itch [7,8]. The interplay between psychological distress and the progression of pruritic conditions is mediated by an affective dimension of itch, which intensifies its perception and drives maladaptive behaviors like scratching [3,9,10]. This vicious itch–scratch cycle not only aggravates underlying skin conditions, but also increases the risk of developing psychiatric comorbidities, such as depression and anxiety, or even suicidal ideation [10,11,12].

Although psychological factors significantly affect the onset, intensity, and progression of inflammatory skin diseases, they are often overlooked in clinical practice [13]. Studies have shown that psychodermatological treatment approaches are effective in reducing pruritus and improving patients’ overall well-being, highlighting their importance in managing the biopsychosocial complexity of these conditions [14,15,16]. A narrow dermatological focus often fails to fully address this complexity, emphasizing the critical need for multimodal and interdisciplinary treatment approaches [17,18].

Within the German-speaking population, a significant proportion suffers from chronic inflammatory skin diseases [19,20,21], yet access to psychodermatological care in this region remains alarmingly limited, being confined primarily to a few highly specialized clinics or centers [22]. Consequently, large numbers of patients remain underserved, resulting in suboptimal care and persistent under-treatment.

Addressing these healthcare disparities has become a priority in public health. Telemedicine, as a model of remote healthcare delivery, is increasingly recognized for its potential to bridge these gaps, offering enhanced accessibility and demonstrating outcomes comparable to traditional face-to-face consultations across various fields [23,24,25,26]. Recent studies highlight high levels of patient satisfaction with teledermatology services [27], implicating its promise in expanding the scope of care by integrating psychological support for individuals with chronic inflammatory skin diseases. However, despite its potential, research on the feasibility, acceptability, and efficacy of telemedicine interventions in psychodermatology remains scarce, compounded by methodological limitations and a lack of robust evidence.

This study aims to address these gaps by investigating the unmet needs of patients with chronic inflammatory skin diseases in German-speaking regions and evaluating the role telemedicine could play in addressing these healthcare disparities. Specifically, this research focuses on the psychological burden of pruritus and explores the feasibility of psychotherapeutic interventions via telemedicine in dermatological patients. By examining the intersection of dermatology and psychology, this study seeks to contribute to the development of more integrated, accessible, and patient-centered approaches to care.

## 2. Materials and Methods

### 2.1. Study Context

This study sought to address the psychological burden of pruritus in individuals with chronic inflammatory skin diseases through video consultations conducted by a licensed psychotherapist. Participants were invited to engage in a complimentary consultation session via the newsletter of the Network of People with Autoimmune Diseases (https://www.nik-ev.de, accessed on 8 March 2025). This newsletter follows a campaign week dedicated to chronic inflammatory skin diseases. To maintain a targeted approach, this study specifically included individuals diagnosed with atopic dermatitis, psoriasis, or acne inversa, as these conditions were the central focus of the campaign, which began on 22 April 2024, with an interprofessional online event. This study employed both quantitative data, including information on underlying diseases, associated pruritus, and psychological distress, as well as qualitative data derived from video consultations and subsequent written feedback from participants. In addition, the consultations incorporated psychoeducational content on itch management and introduced participants to simple psychological interventions. These interventions aimed to enhance understanding of the bio-psycho-social dimensions of pruritus, promote relaxation, and demonstrate techniques for attentional control to reduce the perception of itch.

### 2.2. Participants and Recruitment

Participants were recruited through the newsletter of the Network of People with Autoimmune Diseases. Eligible individuals included adults diagnosed with chronic inflammatory skin diseases, specifically atopic dermatitis, psoriasis, or acne inversa. All diagnoses were revealed by a board-certified dermatologist via the teledermatology platform derma2go (https://derma2go.com, accessed on 8 March 2025). No exclusion criteria were applied apart from the requirement for access to a stable internet connection. Participation in this study was entirely voluntary and offered free of charge. The patients were asked via email if the collected data could be anonymized and used for scientific purposes. The online consultation sessions took place between 3 May 2024 and 3 June 2024. As a token of appreciation, all participants received a personalized summary of the key points discussed during their consultation. This summary included tailored recommendations for suitable exercises and additional information to support their itch management and psychological well-being.

### 2.3. Data Collection

Data were collected during structured interviews conducted as part of the video consultations with a licensed psychotherapist. The consultations were facilitated via the platform Sprechstunde.online (https://sprechstunde.online, accessed on 8 March 2025), where participants were able to independently register and book a time slot for their session. Each consultation followed a standardized protocol, including the following elements:

- Diagnosis: Verification of the participant’s chronic inflammatory skin disease diagnosis.
- Symptoms and related limitations: Identification of the most distressing symptoms associated with the condition, along with the accompanying limitations they impose on daily life and social functioning.
- Current therapy: Documentation of ongoing dermatological and psychological treatments.
- Stressors and resources: Assessment of factors contributing to psychological distress as well as available coping resources.

After the video consultation, the participants were invited to respond to an email containing the following four questions:

1. On a scale from 0 (not at all) to 100 (very strongly), how much do you psychologically suffer due to your dermatological condition?
2. Do you anticipate that psychodermatological treatment could influence your dermatological symptoms?
3. How frequently would you prefer psychodermatological treatment (not at all, sporadically, regularly)?
4. Would you prefer an online consultation or an in-person appointment?

### 2.4. Interventions on Itch Management

The video consultations incorporated psychoeducational interventions specifically focused on itch management. These interventions were grounded in a bio-psycho-social itch model to enhance participants’ understanding of the interaction between psychological, social, and dermatological/biological factors. The delivered content is outlined below, along with an explanation of its relevance and centrality in the treatment of pruritus patients.

- Biopsychosocial model of itch: The biopsychosocial model of itch, based on the framework proposed by Verhoeven and colleagues [10], highlights the interaction between biological, psychological, and social factors in the perception of pruritus. While biological processes contribute to itch, psychological factors such as coping strategies, cognitive biases, and social support play a significant role. Psychological stress (e.g., high workload) can lower the threshold for itch perception and exacerbate symptoms. Coping mechanisms and illness beliefs influence how patients respond to stress, either amplifying or alleviating the experience of itch. Scratching, an automatic response, perpetuates the itch–scratch cycle. Physiologically, stress can activate the HPA axis, which may further aggravate itch. However, psychological factors are central to understanding and managing chronic itch [10]. In the joint online session, the development of an individual disease model was initiated.
- Selective/focused attention: Cognition is known to exert significant, bidirectional effects on the perception of itch. For instance, directing attention to bodily sensations can increase the perception of itch, whereas engaging in distraction techniques can alleviate it [28,29]. To enhance the understanding of selective attention, the *Lemon Imagery Exercise* can be performed. This exercise demonstrates how focusing attention can evoke and intensify physical reactions. The effect of this practice can be leveraged to modulate the perception of itch. To support this effect in daily life, techniques such as the use of “skills” (e.g., a spiky ball) or mindfulness exercises that stimulate the senses can be incorporated.
- Self-efficacy: In chronic pruritus, the cognitive strategies and coping mechanisms employed by patients to manage anxiety and stress significantly influence itch perception and scratching behavior. Negative factors such as resignation, low self-efficacy, or a lack of resilience may exacerbate itch intensity [30], whereas a sense of control and a proactive attitude have been associated with lower pruritus severity and reduced frequency of itching [31,32]. By specific relaxation techniques (e.g., breathing exercises, progressive muscle relaxation), individuals can learn to reduce their stress levels. The experience of being able to exert control strengthens feelings of self-efficacy and reduces feelings of hopelessness. At the same time, the review by Yosipovitch and colleagues highlights that multimodal approaches can help interrupt the itch–scratch cycle in atopic dermatitis [33], with relaxation therapies potentially reducing the urge to scratch.

### 2.5. Data Analysis

All collected data were systematically organized in an Excel spreadsheet for analysis. Quantitative data, such as ratings of psychological burden and preferences for psychodermatological treatment, were analyzed descriptively. Frequencies, averages, and standard deviations were calculated to identify trends and patterns. Qualitative data from written responses and session notes were analyzed using thematic analysis to identify recurring patterns.

## 3. Results

### 3.1. Characteristics of Participants

A total of five female participants, with a mean age of 40.4 years (SD = 11.71), took part in psychodermatological video consultations (Table 1). Four of them were diagnosed with psoriasis, and one with atopic dermatitis (neurodermatitis), both conditions associated with itching. All participants had experienced symptoms for over a year, with symptom onset or exacerbation linked to stressful events.

All participants were under dermatological care and received topical and/or systemic therapy as prescribed by their dermatologist. However, none felt sufficiently treated by dermatology alone. Reported barriers to specialized multimodal treatment included limited awareness of available options, long travel distances, and extended waiting times. Participants were recruited via the Network of People with Autoimmune Diseases. While the newsletter promoting the consultations reached 174 individuals, demographic details of the recipients were unavailable.

### 3.2. Results from the Online Consultations

A total of 174 individuals received the newsletter promoting the psychodermatological video consultations. Of these, 124 opened the newsletter, and 16 clicked the link to schedule a consultation. Five individuals ultimately participated in online consultations over one month (3 May 2024 and 3 June 2024) (Table 2).

Three out of the five participants reported experiencing pruritus, with an average intensity of 75/100 on a Visual Analog Scale (VAS) ranging from 0 to 100 (SD = 22). Two participants reported no pruritus. All participants who experienced pruritus identified a connection between their symptoms and psychological factors. The most frequently reported additional symptoms were the following:

- Sleep disturbances due to itching, which significantly impacted daily functioning.
- Reduced ability to relax in everyday life, caused by a combination of existing daily stressors (e.g., high work demands) and ongoing health-related issues (e.g., focusing on a distressing symptom or the consideration of additional treatment options). According to participants’ feedback, this reduced relaxation ability prevented them from engaging in activities that would typically provide relief or promote well-being.
- Discomfort in their own bodies, which primarily led to increased uncertainties in social interactions. Participants reported spending significant time on concealing their condition, which affected their confidence and social engagement.

### 3.3. Results from the Follow-Up Surveys via Email

Following the online consultations, four of the five participants completed a written follow-up survey (Table 3). Respondents reported an average distress level of 74/100 on the VAS (SD = 16). All anticipated a positive impact of psychodermatological treatment on their dermatological symptoms. Regarding treatment preferences, the following was found:

- Frequency: Three preferred regular treatment (approximately every two weeks), particularly in the initial phase, with longer intervals over time. One participant sought more information before deciding.
- Modality: Two preferred online consultations, one rated online and in-person consultations equally, and one preferred in-person consultations.

## 4. Discussion

This study aimed to assess the need for psychodermatological services for patients with chronic inflammatory skin diseases in German-speaking countries and to evaluate telemedicine as a potential solution to existing care gaps. Focusing on pruritus, a common and distressing symptom influenced by psychological factors, this study highlights telemedicine as a promising approach to improving access to psychodermatological care. However, the results are preliminary and need further investigation.

### 4.1. The Psychological Burden of Pruritus

Pruritus is a highly distressing symptom for patients with chronic inflammatory skin diseases, severely impairing quality of life. Beyond physical discomfort, it often leads to sleep disturbances, anxiety, social isolation, and reduced functionality [11,12], as partly also shown in our findings. Despite these significant impacts, the psychological burden associated with pruritus is frequently overlooked in clinical practice [13]. This study suggests that psychological stress not only results from the disease, but also contributes to its onset and progression, as reported from all five patients.

Affective factors such as anxiety, depression, and stress intensify pruritus and perpetuate the itch–scratch cycle, worsening dermatological symptoms [3,9,10]. Heightened symptom focus amplifies distress, hindering participation in soothing activities, which was also described by our participants. This aligns with the theory of attentional focus, linking symptom-centered perception to increased psychological distress [28,29].

Participants rated their psychological burden at 74/100 on the VAS, underscoring the severity of their impairment and the limitations of traditional dermatological treatments. While therapies like topical agents, systemic drugs, and phototherapy are central to managing these conditions, they fail to address the psychological and emotional dimensions. Additionally, this study identified an association between stressful life events and disease onset or exacerbation, reinforcing the bidirectional relationship between psychological stress and skin conditions, as shown in previous studies [7,8].

Participants expressed a clear need for psychodermatological care, hoping it would improve both disease outcomes and overall well-being. This highlights a critical gap in the healthcare system and emphasizes the importance of integrated, interdisciplinary treatment approaches combining dermatological and psychological interventions.

### 4.2. The Psychodermatological Care Gap

This study suggests that psychodermatological care in German-speaking countries may be insufficient. Despite growing evidence of the psychological factors influencing chronic inflammatory skin diseases, specialized services appear to be limited to a few clinics, potentially leading to long wait times and geographic barriers [22]. Most participants reported inadequate information about psychodermatological care, with some unaware of its relevance. These findings align with previous research highlighting disparities in psychodermatological service availability across Europe. While Western Europe and Scandinavian countries provide relatively good access to such care, availability remains limited in Eastern, Southeastern, and some Southern and Central European regions. Strengthening the integration of psychodermatological approaches across all European regions is essential to ensure more comprehensive care [22].

The limited integration of dermatology and psychology in clinical practice presents a significant challenge to optimizing treatment outcomes. Traditional dermatological therapies, while essential, often overlook the emotional and psychological dimensions of pruritus, potentially leading to suboptimal results. Awareness among dermatologists regarding the benefits of interdisciplinary collaboration appears to be limited. Psychodermatological treatments tend to be more accessible at university hospitals and specialized centers, likely due to their proximity to research and interprofessional resources, whereas availability in smaller private practices remains scarce [22].

Telemedicine offers a promising solution to address these gaps by facilitating interdisciplinary collaboration, increasing awareness of psychodermatological care options, and expanding service accessibility. Studies have demonstrated the effectiveness of psychodermatological treatments, showing significant improvements in both dermatological symptoms and psychological well-being, which further supports their integration into telemedicine models [14,15,16]. A multidisciplinary approach could not only enhance care delivery, but also improve cost efficiency by enabling the comprehensive management of both dermatological and psychological conditions [34]. Our study suggests patient interest in telemedicine, aligning with research indicating comparable satisfaction and treatment efficacy between telemedicine and in-person consultations [25,26].

### 4.3. Telemedicine in Psychodermatology

This study suggests telemedicine as a promising tool for overcoming barriers in psychodermatological care. In line with recent research [35], which explores the integration of psychodermatology into telemedicine, our findings support the potential of telemedicine in improving access to psychodermatological services and enhancing patient outcomes. Participants expressed optimism about the positive effects of psychodermatological treatment on their chronic inflammatory skin diseases and associated symptoms. The preference for telemedicine aligns with studies reporting high patient satisfaction with telemedicine services in dermatology [27].

Telemedicine offers several advantages, including improved accessibility and the ability to reach underserved populations. Patients in rural or remote areas and those with limited mobility can benefit significantly, reducing geographical and physical barriers [36]. Telemedicine also facilitates stronger interdisciplinary collaboration, allowing dermatologists, psychotherapists, and other specialists to develop comprehensive and individualized treatment plans. This collaborative approach is particularly valuable for managing chronic inflammatory skin diseases, where complex interactions among dermatological, psychological, and social factors necessitate close cooperation [18].

Despite these benefits, challenges remain. The digital divide, including inequalities in access to technology and digital literacy, poses a significant barrier. Older adults and those in areas with limited internet access may be excluded [37]. To address this, telemedicine platforms should be user-friendly and accessible to individuals with varying technological expertise. Additionally, training and support for healthcare providers are essential. Telemedicine requires different skills and workflows compared to traditional consultations, making training in remote care delivery critical for ensuring high-quality services [38].

The implementation of telemedicine requires a substantial time investment, not only for training, but also for adapting existing workflows. Furthermore, there is a concern that online consultations could potentially replace or diminish the availability of face-to-face consultations, which are often crucial for establishing trust and building therapeutic relationships [39]. Another critical aspect is the exchange of information among healthcare providers. For effective interdisciplinary collaboration, it must be clarified whether shared patient records will be used, or if additional time will need to be allocated for communication and coordination between dermatologists, psychotherapists, and other specialists.

These open questions highlight the need for careful planning during implementation. Addressing these challenges will be essential to ensure that telemedicine can complement, rather than compete with, traditional care approaches and facilitate seamless collaboration among professionals while maintaining high standards of patient care.

### 4.4. Limitations, Future Research, and Implications

This study provides a foundation for future research in psychodermatology and telemedicine. While the findings offer valuable insights into the potential benefits of telemedicine interventions, the small sample size and the limited geographic scope highlight the need for larger and more diverse studies. Future research should aim to increase the sample size to improve the reliability and generalizability of the results. Additionally, the recruitment method may have introduced selection bias, as participants self-enrolled via a link, likely attracting individuals already interested in or optimistic about such services. This may have led to an over-representation of those with favorable attitudes toward psychodermatological care and telemedicine, potentially skewing the findings. A more objective approach, such as random selection or recruitment through dermatology clinics, could help mitigate this bias. Moreover, incorporating a control group in future studies would provide a clearer understanding of whether telemedicine consultations yield significantly different outcomes compared to traditional dermatological care. Expanding the research scope to include a broader range of pruritic conditions is also crucial. While this study focused on three specific chronic inflammatory skin diseases, pruritus is a common symptom across various dermatological conditions. A more comprehensive assessment of different itch patterns and their psychological impact could further refine the understanding of psychodermatological interventions.

Future research should assess the long-term effectiveness of telemedicine-based psychodermatological interventions, focusing on their impact on symptom reduction, quality of life, and patient satisfaction. Further investigation is needed to explore the feasibility and efficacy of telemedicine across diverse cultural and healthcare settings and to identify potential implementation barriers.

Future studies could also examine the specific psychological interventions most effective for patients with chronic inflammatory skin diseases and pruritus. While this study included psychoeducational content on itch management, attention regulation, and self-efficacy strategies, evaluating the comparative efficacy of therapeutic approaches like Cognitive Behavioral Therapy (CBT) or Acceptance and Commitment Therapy (ACT) would be valuable. Investigating the impact of additional disciplines, such as nutritional counseling or cosmetology, on treatment outcomes and patient satisfaction could also provide valuable insights. 

## 5. Conclusions

In summary, this study underscores the importance of addressing psychological factors in the onset and progression of chronic inflammatory skin diseases, particularly regarding pruritus. Telemedicine emerges as a promising tool to improve access to psychodermatological care. Integrating psychological support into dermatological treatment through telemedicine holds potential for enhancing outcomes and bridging care gaps. However, further research is necessary to refine telemedicine interventions and evaluate their widespread implementation. The small sample size in this study emphasizes the need for future studies to include a larger number of participants. The psychoeducational strategies for itch management outlined in Section 2.4 could already be incorporated into routine dermatological practice to enhance psychodermatological patient care.

## Figures and Tables

**Table 1:** Characteristics of participants.

Features	Total Sample		
	*n*	M	SD
Total participants	5		
Age		40.4	11.71
Sex			
Female	5		
Male	0		
Skin disorder			
Psoriasis	4		
Atopic dermatitis	1		
Symptom duration			
>1 year	5		
<1 year	0		
Dermatological care	5		
Psychological care	0		
Sufficiently treated by dermatology alone	0		

**Table 2:** Results from the online consultation.

Features	Total Sample		
	*n*	M	SD
Newsletter recipients	174		
Opened newsletter	124		
Clicked on consultation link	16		
Participated in video consultation	5		
Experienced pruritus	3		
Average pruritus intensity (0–100 VAS)		75	22
Identified a psychological connection	3		
Sleep disturbances due to itching	3		
Reduced ability to relax	5		
Discomfort in social interactions	5		

**Table 3:** Results from the follow-up surveys via email.

Features	Total Sample		
	*n*	M	SD
Participants who were contacted	5		
Participants who competed the follow-up survey	4		
Distress level due to dermatological condition (0–100 VAS)		74	16
Anticipated positive impact of psychodermatological treatment			
Yes	4		
No	0		
Preferred frequency of treatment			
Regular treatment	3		
No answer	1		
Preferred treatment modality			
Online	2		
Both online and in-person	1		
In-person	1		

## Data Availability

Due to privacy/patient confidentiality data is not public.
